# Chronic vagus nerve stimulation is associated with multi-year improvement in intrinsic heart rate recovery and left ventricular ejection fraction in ANTHEM-HF

**DOI:** 10.1007/s10286-021-00780-y

**Published:** 2021-02-16

**Authors:** Bruce D. Nearing, Imad Libbus, Gerrard M. Carlson, Badri Amurthur, Bruce H. KenKnight, Richard L. Verrier

**Affiliations:** 1 Department of Medicine, Division of Cardiovascular Medicine, Beth Israel Deaconess Medical Center, Harvard Medical School, 99 Brookline Avenue, RN-301, Boston, MA 02215-3908 USA; 2 LivaNova USA, Houston, TX USA

**Keywords:** Autonomic regulation therapy, Baroreflex sensitivity, Heart failure, Heart rate recovery, Heart rate turbulence, Vagus nerve stimulation

## Abstract

**Purpose:**

Disturbed autonomic function is implicated in high mortality rates in heart failure patients. High-intensity vagus nerve stimulation therapy was shown to improve intrinsic heart rate recovery and left ventricular ejection fraction over a period of 1 year. Whether these beneficial effects are sustained across multiple years and are related to improved baroreceptor response was unknown.

**Methods:**

All patients (*n = *21) enrolled in the ANTHEM-HF clinical trial (NCT01823887, registered 4/3/2013) with 24 h ambulatory electrocardiograms at all time points and 54 normal subjects (PhysioNet database) were included. Intrinsic heart rate recovery, based on ~ 2000 spontaneous daily activity-induced heart rate acceleration/deceleration events per patient, was analyzed at screening and after 12, 24, and 36 months of chronic vagus nerve stimulation therapy (10 or 5 Hz, 250 μs pulse width, 18% duty cycle, maximum tolerable current amplitude).

**Results:**

In response to chronic high-intensity vagus nerve stimulation (≥ 2.0 mA), intrinsic heart rate recovery (all time points, *p* < 0.0001), heart rate turbulence slope, an indicator of baroreceptor reflex gain (all, *p* ≤ 0.02), and left ventricular ejection fraction (all, *p* ≤ 0.04) were improved over screening at 12, 24, and 36 months. Intrinsic heart rate recovery and heart rate turbulence slope were inversely correlated at both screening (*r* = 0.67, *p* < 0.002) and 36 months (*r* = 0.78, *p* < 0.005).

**Conclusion:**

This non-randomized study provides evidence of an association between improvement in intrinsic heart rate recovery and left ventricular ejection fraction during high-intensity vagus nerve stimulation for a period of ≥ 3 years. Correlated favorable effects on heart rate turbulence slope implicate enhanced baroreceptor function in response to chronic, continuously cyclic vagus nerve stimulation as a physiologic mechanism.

## Introduction

Heart failure with reduced left ventricular ejection fraction (LVEF) (HFrEF) constitutes a chronically deteriorating condition resulting in increased morbidity and mortality. HFrEF is responsible for an estimated 352,000 deaths annually in the United States alone [1]. Although many forms of pharmacologic and device therapy have evolved, the success has been moderate in ameliorating the progression of HFrEF. Disturbances in autonomic function have been recurrently implicated, manifest as excess sympathetic nerve activity and decreased cardiac vagal tone [2]. Vagus nerve stimulation (VNS) has been pursued as a mode of therapy to improve autonomic balance [3]. A distinct advantage of VNS is its safety profile, documented in the management of > 120,000 patients with epilepsy or depression over the span of > 30 years [4, 5]. It is noteworthy that the protective action of chronic VNS against seizure in drug-resistant epilepsy persists for more than a decade [6, 7].

In their pioneering work, Schwartz, De Ferrari, and their coworkers [8] reported improved LVEF and New York Heart Association (NYHA) class in a first-in-human study. The investigators employed the CardioFit™ system (BioControl, Yehud, Israel), which consisted of an implantable neurostimulator device that delivered low-current impulses to the right cervical vagus nerve. A unique feature was that the stimulator was designed to sense heart rate via an electrode in the right ventricle to help regulate VNS and to avoid excess bradycardia while delivering effective stimulation. These observations were further supported by results of the multicenter international CardioFit study [9]. Disappointingly, the Increase Of VAgal TonE in Heart Failure (INOVATE-HF) trial, a large follow-up investigation that enrolled more than 700 patients, was terminated early due to futility in meeting the primary endpoint of reduction in all-cause mortality or first heart failure hospitalization events, notwithstanding substantial improvements in secondary endpoints (NYHA class, quality of life, and 6-minute walk test) [10]. Post hoc analysis of the INOVATE-HF study disclosed that sufficient stimulation levels were not attained among all patients and that a criterion response to VNS was observed in only 30% of patients [11]. By comparison, the Autonomic Neural Regulation Therapy to Enhance Myocardial Function in Heart Failure (ANTHEM-HF) interventional clinical trial utilized a stimulation protocol associated with significant improvements in cardiac mechanical function and heart failure symptoms with a desirable safety profile [12]. In this study, appropriate VNS dosing was assessed in all patients during a titration period following implant of the neural stimulation system.

Recently, using a novel approach, Carlson et al. [13] found in 24 h ambulatory ECG (AECG) recordings of patients enrolled in the ANTHEM-HF study, that VNS improved spontaneous intrinsic heart rate recovery (HRR) over a 1-year period compared to screening. In individual patients, ~ 2000 heart rate acceleration/deceleration events during daily activity were analyzed. The primary parameter measured was the time constant of the dynamics of the native spontaneous HRR. The improvement in intrinsic HRR was associated with beneficial effects on LVEF. These observations are particularly relevant in light of extensive evidence that HRR upon cessation of exercise tolerance testing is a powerful and independent predictor of cardiovascular mortality and sudden cardiac death [14]. McCrory and coworkers [15] determined in a nationally representative > 4000-subject sample of individuals older than 50 years that the temporal rate of HRR in response to an orthostatic challenge is a highly predictive indicator of mortality risk.

The putative mechanistic basis for the capacity of intrinsic HRR to predict cardiac events is that this parameter reflects the gain of the baroreceptor reflex [14, 16]. Baroreceptor reflex function can be assessed in humans by injection of a pressor agent such as phenylephrine and measuring the regression slopes between arterial blood pressure and RR intervals [17]. An evolution of this approach is the measurement of heart rate turbulence (HRT), which can be monitored by measuring the RR intervals following spontaneous ventricular premature beats in AECG recordings and has been shown in numerous studies to be highly predictive of cardiovascular mortality and to correlate with baroreceptor reflex function [18].

The main goal of the present study was to determine whether VNS therapy is associated with improvements in intrinsic HRR and LVEF at 24 and 36 months. The secondary aim was to determine whether the enhancement in intrinsic HRR is linked to baroreceptor sensitivity. To evaluate this hypothesis, we examined the correlation between intrinsic HRR and HRT slope at screening and after 36 months of chronic VNS.

## Methods

### Patient data and characteristics

The study design and patient selection criteria of the ANTHEM-HF interventional clinical trial (NCT01823887, registered 4/3/2013) were previously described [19]. The investigation, which complied with the Declaration of Helsinki, was conducted in Secunderabad, Ahmedabad, Haryana, and Goa, India. Local ethics committees at all sites approved the protocol, and all patients provided written informed consent in local languages. The present sub-study was conducted under a protocol approved by Beth Israel Deaconess Medical Center’s (BIDMC’s) Institutional Review Board.

Briefly, 60 patients with NYHA class II/III heart failure and age ≥ 18 years were enrolled at 10 sites. Inclusion criteria were LVEF ≤ 40%, left ventricular end-diastolic dimension ≥ 50 and < 80 mm, QRS complex width ≤ 150 ms, and receiving optimal medical management. Specifically, this included stable beta-blocker therapy for heart failure as indicated for at least 3 months and all other oral pharmacologic therapy for heart failure, such as angiotensin-converting enzyme inhibitors or angiotensin receptor blockers, loop diuretics, and spironolactone, for at least 1 month. No changes in heart failure medications were made during the study. Patients needed to be able and willing to perform a 6-minute walk test with a distance of 150–425 meters at screening, limited by heart failure symptoms.

All ANTHEM-HF patients received a VNS system (LivaNova USA, Inc., Houston, TX, USA) on either the left or the right cervical vagus nerve. VNS was titrated to the maximum tolerable intensity over the course of 10–12 weeks. Chronic cyclic stimulation was delivered at a frequency of 10 Hz for 12 months and at a frequency of 5 Hz after 24 months, a pulse width of 250 μs, and a duty cycle of 14 s on/66 s off. Continuous cyclic stimulation was sustained as tolerated for the entire titration period and the 36-month follow-up period; for 8 patients, the maximum tolerable stimulation current amplitude was < 2 mA (low-intensity), and for 13 patients, amplitude was ≥ 2 mA (high-intensity).

In 25 ANTHEM-HF patients, AECGs (DigiTrak XT, Philips Medical Systems, Best, The Netherlands) were recorded at screening and after 12, 24, and 36 months of chronic therapy. Four patients had excess premature ventricular beats that precluded intrinsic HRR analysis; the remaining 21 patients provided the data set for the present analysis during the 3-year follow-up.

De-identified 24 h AECG recordings from 54 normal subjects during normal daily activities (30 men, age 28.5–76 years; 24 women, age 58–73 years) were acquired from the publicly available PhysioNet Normal Sinus Rhythm database [20]. These subjects were in normal sinus rhythm and were healthy with respect to disease history, physical examination, and 12-lead ECG, and were taking no drugs [21]. The designation “healthy” denotes not only the absence of coronary heart disease but also the absence of any other diseases that can be detected by these tests.

### Intrinsic heart rate recovery

**Fig. 1:**
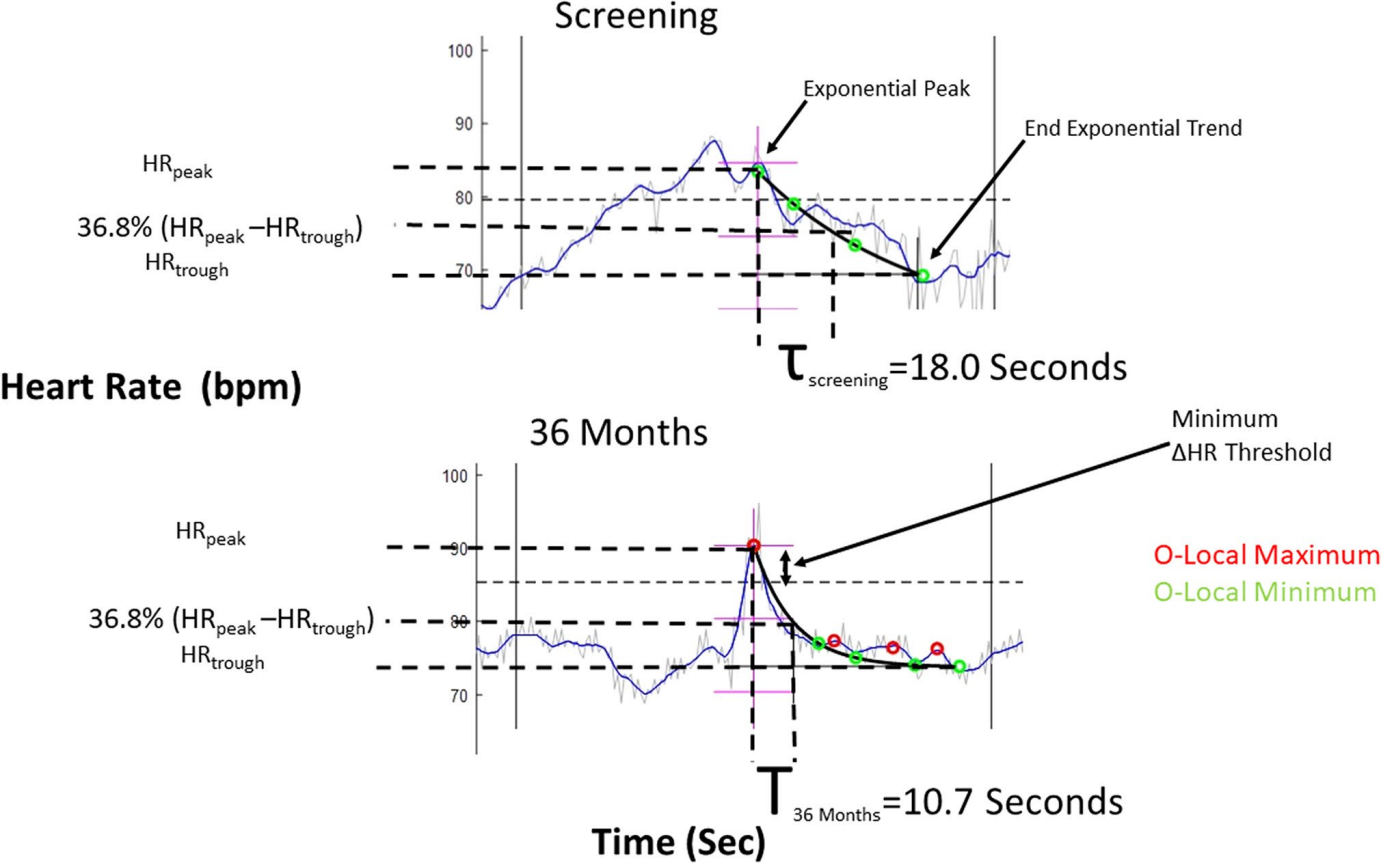
Comparison of the pattern of intrinsic heart rate recovery (HRR) based on spontaneous heart rate changes in ambulatory electrocardiograms (AECGs). Essentially, an initial heart rate (HR) maximum point (HR_peak_) is identified, and subsequent HRR to the final heart rate (HR_trough_) is fit to a mono-exponential decay with time constant of recovery (tau), based on a sequence of smoothed and decaying heart rate maxima and minima, as described in detail in the methods section. The *x*-axis represents a portion of the 24 h monitoring period, and the *y*-axis displays the patient’s heart rate. In a representative patient, VNS reduced tau by 40.6%, from 18.0 s at screening (upper panel) to 10.7 s at 36 months of therapy (lower panel)

As previously described [13], intrinsic HRR was calculated as the slope in seconds of the gradual decrease in heart rate following a spontaneous heart rate surge, such as routinely occurs in association with daily activities such as postural changes, emotions, and physical exertion [15, 16, 22]. Intrinsic HRR was measured in terms of mean banded relaxation parameter by first finding the peaks and troughs in the heart rate trend for each 24 h recording (Fig. 1). Instantaneous heart rate data points were smoothed to eliminate isolated spikes in heart rate changes. Paired local maxima and local minima of heart rate data were identified and analyzed to determine the candidate intrinsic HRR events in the heart rate stream. A first-order exponential was derived for each identified intrinsic HRR event to produce a time constant (tau) and a corresponding change in heart rate from the first maximum and the subsequent minima.

The exponential time constant (tau) for each exponential fit was put into separate bins according to the magnitude of the heart rate surge within a window of ±8 beats. The tau values for each bin were averaged and combined into a mean banded relaxation parameter. A lower mean banded relaxation parameter indicates a steeper recovery, and a shorter time constant of recovery indicates a more rapid intrinsic HRR.

Specifically, for quantification of intrinsic HRR, heart rate deceleration was modeled as a mono-exponential decay at multiple time points, where the time constant (tau) indicates heart rate decay. The equation of the trend line for this decay is:

$${\text{HR}}\;(t)\; = \;a0\; + \;b \cdot e\;( - 1/Tau) \cdot t$$

In this equation, *a*0 is the asymptotic value of the heart rate, *b* is the decremented value from the peak heart rate, and tau is the time constant, which reflects intrinsic HRR such that a lower value of the time constant reflects a more rapid intrinsic HRR response.

### Heart rate turbulence

HRT was evaluated according to the method developed and standardized by Schmidt, Bauer, and coworkers, who reviewed supporting literature in a consensus report [18]. HRT onset, which calculates the initial brief acceleration in sinus rate after a premature ventricular contraction, and HRT slope, which measures the subsequent heart rate deceleration, were evaluated as continuous variables. HRT onset of ≥ 0% and HRT slope of ≤ 2.5 ms per RR interval have been determined to be abnormal [18].

### Left ventricular ejection fraction

Echocardiograms were recorded with standard transthoracic echocardiographic equipment and LVEF was calculated using the biplane method. The recordings were coded and de-identified of patient and sample information and sent to designated core laboratory facilities for analysis. LVEF ≤ 35% is considered indicative of stable chronic heart failure [23].

### Statistical methods

Results were analyzed using SAS 9.4 (SAS Institute, Cary, NC, USA), which allowed verification of the normality of the data. Analyses of patient parameters in Table 1 were performed with *t* tests for continuous variables and with Fisher exact test for discrete variables. Effects of VNS on intrinsic HRR and other parameters presented in Table 2 were analyzed with paired *t* tests with Bonferroni correction for multiple comparisons. The SAS mixed linear model was used to evaluate the covariance of LVEF, HRT slope, heart rate, and the mean banded relaxation parameter. Data are reported as means ± SEM. *p* < 0.05 was considered statistically significant.

**Table 1:** Patient characteristics at screening

Demographics	Full cohort (*n* = 21)	Low-intensity VNS (*n* = 8)	High-intensity VNS (*n* = 13)	Significance^a^
Age (years)	46.2 ± 2.4	42.9 ± 4.3	48.3 ± 2.9	0.29
Male, *n* (%)	17 (81)	7 (88)	10 (77)	0.55
Duration of HF (years)	3.7 ± 0.8	3.1 ± 1.4	4.0 ± 1.0	0.59
Ischemic heart failure, *n* (%)	15 (71)	6 (75)	9 (69)	0.78
Nonischemic heart failure, *n* (%)	6 (29)	2 (25)	4 (31)	0.25
NYHA class II/III, *n* (%)	13 (62)/8 (38)	4 (50)/4 (50)	7 (54)/6 (46)	0.86
MLHFQ score	42.4 ± 2.6	42.5 ± 3.5	42.5 ± 3.7	0.99
Body mass index (kg/m^2^)	23.8 ± 0.8	25.0 ± 1.4	23.0 ± 1.0	0.24
LVEF (%)	32.5 ± 1.5	32.4 ± 2.5	32.6 ± 2.0	0.94
LVESV (mL)	102.7 ± 6.7	102.3 ± 8.4	103.0 ± 9.8	0.96
LVESD (mm)	51.3 ± 1.3	51.9 ± 2.0	50.9 ± 1.7	0.72
LVEDV (mL)	150.9 ± 8.2	150.5 ± 8.5	151.2 ± 12.5	0.97
LVEDD (mm)	61.5 ± 1.1	62.3 ± 1.9	61.1 ± 1.5	0.63
Heart rate (beats/min)	77 ± 2	84 ± 6	78 ± 3	0.32
Systolic BP (mmHg)	106 ± 3	105 ± 5	106 ± 5	0.81
Diastolic BP (mmHg)	72 ± 2	70 ± 3	74 ± 4	0.48
6MWT (meters)	304 ± 13	298 ± 31	302 ± 10	0.89
QRS complex width (msec)	110 ± 5	105 ± 8	112 ± 6	0.49

*6MWT* 6-minute walk test, *BP* blood pressure, *HF* heart failure, *LVEDD* left ventricular end-diastolic dimension, *LVEDV* left ventricular end-diastolic volume, *LVEF* Left ventricular ejection fraction, *LVESD* left ventricular end-systolic dimension, *LVESV* left ventricular end-systolic volume, *MLHFQ* Minnesota Living with Heart Failure Questionnaire, *NYHA* New York Heart Association

^a^Comparing patients receiving low- vs. high-intensity VNS

**Table 2:** Intrinsic heart rate recovery, heart rate, heart rate turbulence, and left ventricular ejection fraction at screening and 12, 24, and 36 months

Measurement	Screening (*n* = 21)	12 months (*n* = 21)	*p* value*	24 months (*n* = 16)	*p* value*	36 months (*n* = 14)	*p* value*
IHRR (s) in patients with low-intensity stimulation	13.0 ± 1.0	13.1 ± 0.1	0.63	12.4 ± 0.1	0.05	12.1 ± 0.1	0.05
IHRR (s) in patients with high-intensity stimulation	12.3 ± 0.1	11.2 ± 0.1	< 0.0001	10.9 ± 0.1	< 0.0001	10.3 ± 0.1	< 0.0001
Heart rate (bpm) in patients with low-intensity stimulation	78.8 ± 3.5	68.4 ± 2.7	0.015	66.5 ± 1.6	0.008	68.0 ± 2.2	0.05
Heart rate (bpm) in patients with high-intensity stimulation	75.9 ± 2.6	67.4 ± 2.9	0.005	66.5 ± 1.5	0.01	65.0 ± 2.3	0.008
HRT slope in patients with low-intensity stimulation (ms/RR interval)	4.8 ± 1.6	6.0 ± 1.6	0.08	6.8 ± 2.3	0.17	7.7 ± 0.9	0.21
HRT slope in patients with high-intensity stimulation (ms/RR interval)	4.6 ± 1.3	9.7 ± 2.2	0.003	8.9 ± 2.0	0.02	9.2 ± 1.7	0.02
LVEF (%) in patients with low-intensity stimulation	32.4 ± 2.5	34.9 ± 2.7	0.21	36.4 ± 2.8	0.04	38.4 ± 2.9	0.1
LVEF (%) in patients with high-intensity stimulation	32.6 ± 2.0	38.7 ± 2.0	0.00828	38.9 ± 2.0	0.04	43.8 ± 2.9	0.0096

*HRT* heart rate turbulence, *IHRR* intrinsic heart rate recovery, *LVEF* left ventricular ejection fraction

*Compared to screening

## Results

The characteristics at screening of the ANTHEM-HF patients whose data were included in the analyses are summarized in Table 1. There were no significant differences at enrollment in characteristics of patients who received low- versus high-intensity VNS therapy. None of the patients had received an implantable cardioverter defibrillator (ICD) or cardiac resynchronization therapy (CRT) device.

### Intrinsic heart rate recovery

**Fig. 2:**
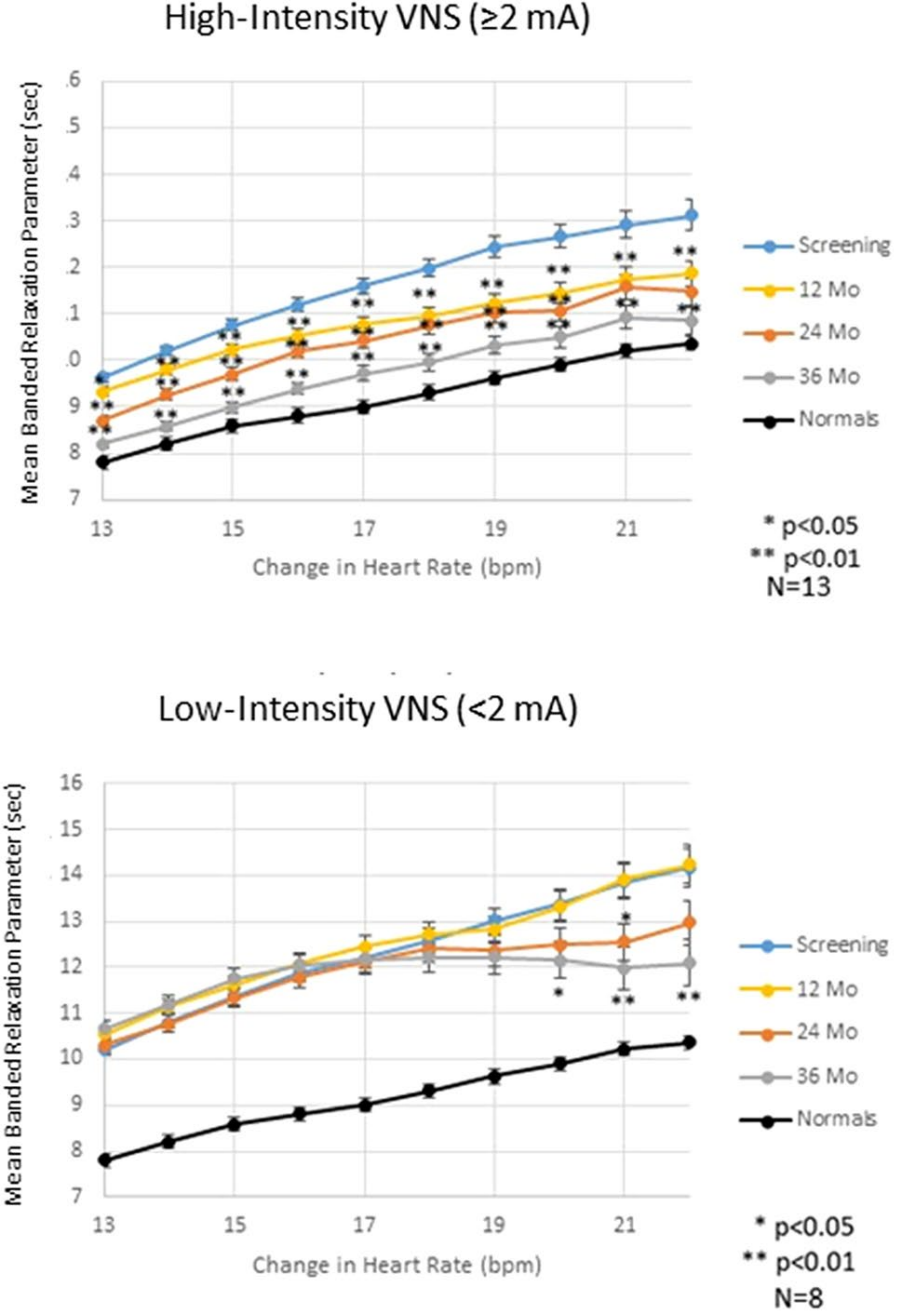
Intrinsic heart rate recovery (HRR) was calculated based on the mean banded relaxation parameter (*y*-axis) for several heart rate changes (*x*-axis) among normal subjects (solid line) and in patients with heart failure at screening (blue line) and after 12 (gold line), 24 (red line), and 36 months (gray line) of vagus nerve stimulation (VNS). Patients with high-intensity VNS (upper panel) showed significant improvements at every change in heart rate compared to screening (*p < 0.05; **p < 0.01), but those with low-intensity VNS (lower panel) showed changes in intrinsic HRR at only a few measurement points

Intrinsic HRR increased consistently as the VNS therapy progressed across the 3-year period studied. The effect was stronger in heart failure patients with high-intensity stimulation (≥ 2 mA) but also occurred in patients with low-intensity stimulation. This effect was reflected as a decrease in the mean banded relaxation parameters for different levels of heart rate change. Mixed-model analysis showed that this effect was independent of the reduction in average heart rate observed in these patients (Table 2) across the 3-year period (*p* = 0.66) (Fig. 2). At screening, subjects with heart failure exhibited a significantly prolonged intrinsic HRR (from 9.8 ± 0.08 to 13.5 ± 0.26 s) as compared to normal subjects (from 7.8 ± 0.04 to 10.4 ± 0.09 s, *p* < 0.005). In patients with high-intensity VNS (≥ 2 mA), intrinsic HRR was improved at the 12-, 24-, and 36-month determinations by 8.9%, 11.4%, and 16.3%, respectively (all *p* < 0.0001 compared to screening) (Table 2), consistent with increased parasympathetic nerve activity. Patients receiving low-intensity stimuli (< 2 mA) did not exhibit intrinsic HRR improvement at 12 months (*p* = 0.63), but the intrinsic HRR response at 24 months was increased by 4.6% and at 36 months by 6.9% (both *p* < 0.05) compared to screening. This effect was one half of that observed in patients with high-intensity VNS (Table 2). Response to VNS therapy in a representative patient comparing the screening heart rate trend to that observed at 36 months is presented in Fig. 1. In this example, the time constant of intrinsic HRR (tau) was reduced by 40.6% from screening (at 18.0 s) to 36 months (at 10.7 s).

### Heart rate

**Fig. 3:**
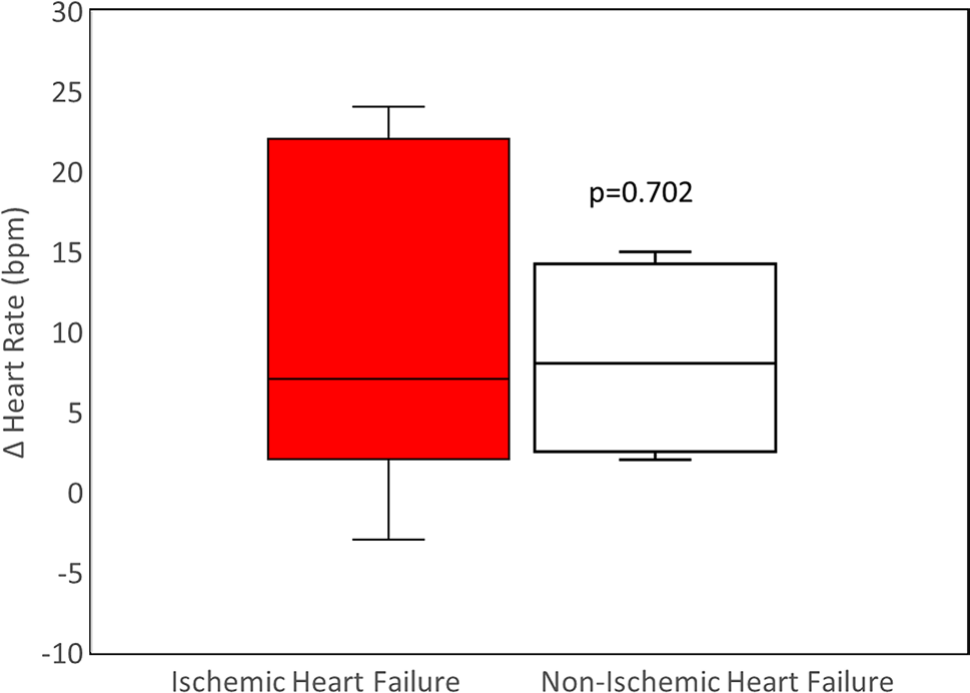
Box-plot comparison by quartiles of heart rate change in response to vagus nerve stimulation in ischemic (*n* = 15, filled red box) and nonischemic heart failure patients (*n* = 6, open box) from enrollment to 36 months. The horizontal bar represents the median. The upper and lower sides of the box represent the upper 75th and lower 25th percentiles, respectively, and the whiskers represent the highest and lowest values. Differences in the distribution of values are not statistically significant (*p* = 0.702)

Heart rate decreased significantly from screening at each time point in both the low- and high-intensity stimulation groups (Table 2). There were no differences in the heart rate response comparing the ischemic and nonischemic groups (*p* = 0.70) (Fig. 3).

### Heart rate turbulence

HRT slope, an indicator of baroreceptor reflex gain, was in the normal range at screening and improved significantly by 110.8% at 12 months, 93.5% at 24 months, and 100.0% at 36 months (all *p* < 0.02) in patients with high-intensity VNS therapy (Table 2). HRT slope was not affected by low-intensity VNS. HRT onset was normal and was not altered by VNS.

**Fig. 4:**
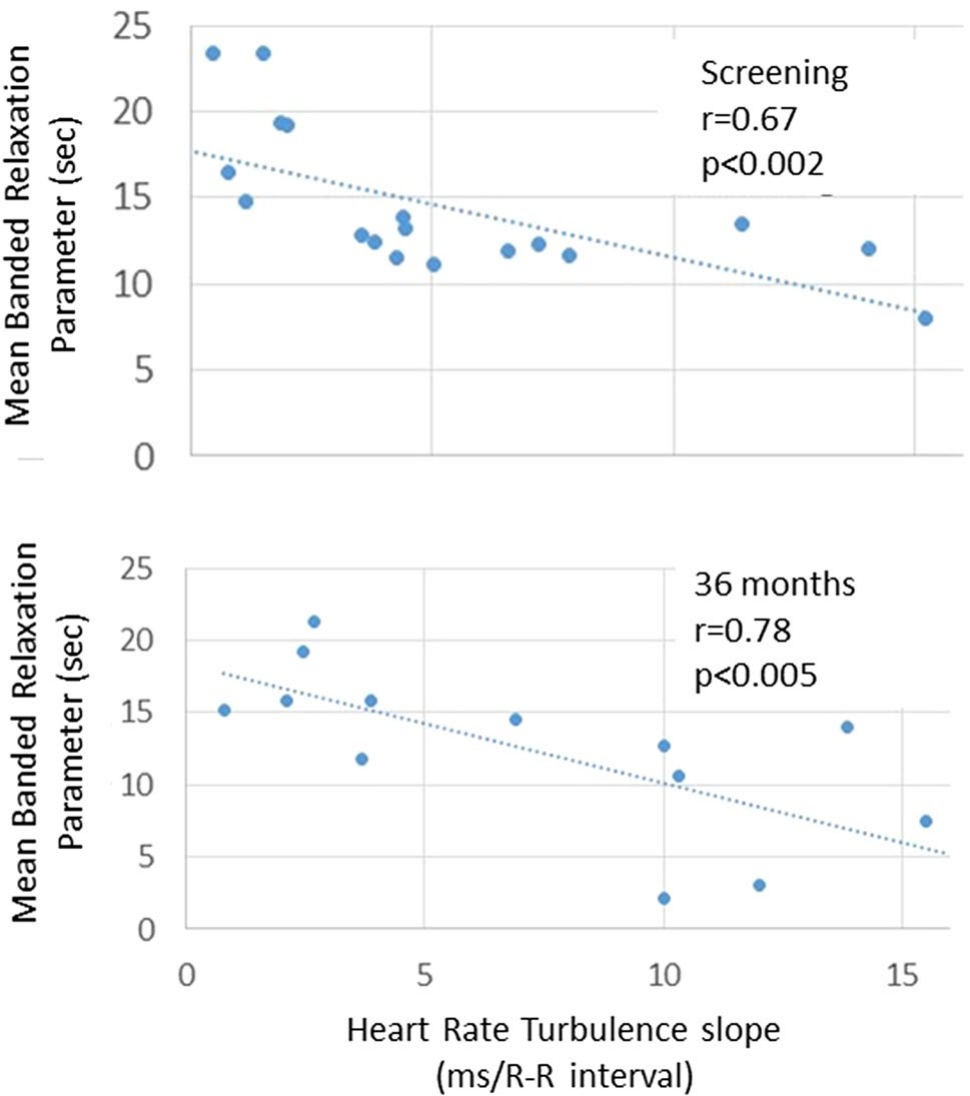
Inverse correlation between mean banded relaxation parameter and heart rate turbulence (HRT) slope. Both at screening (upper panel) and after 36 months of high-intensity vagus nerve stimulation (VNS) (lower panel), there was an inverse correlation between these two parameters, with *r* values of 0.67 (*p* < 0.002) and 0.78 (*p* < 0.005), respectively. These results suggest that acceleration of heart rate recovery (HRR) is linked to increases in HRT slope, consistent with a baroreceptor-mediated modulation of the spontaneous heart rate response to daily activity

Importantly, there was a statistically significant inverse correlation between HRT slope and mean banded relaxation parameter, a measure of intrinsic HRR, at both screening and 36 months following high-intensity VNS (Fig. 4). Thus, the time constant for intrinsic HRR corresponded to the level of baroreceptor sensitivity as reflected in HRT slope. VNS, by increasing vagal tone, reduced the time constant for intrinsic HRR, which is reflected in an augmentation in baroreceptor sensitivity. The relationship between HRT slope and heart rate was not statistically significant (*p* = 0.063).

### Left ventricular ejection fraction

LVEF was improved by high-intensity VNS by 18.7%, 19.3%, and 34.4% at 12, 24, and 36 months, respectively, consistent with a salutary effect on parasympathetic nerve activity (Table 2). By comparison, individuals with low-intensity VNS exhibited significant changes in LVEF only at 24 months (by 12.3%). Mixed-model analysis revealed a statistically significant correlation between increase in HRT slope and improvement in LVEF (p < 0.017).

### Absence of cardiac events

No patient experienced sudden cardiac death, ventricular fibrillation, or sustained ventricular tachycardia during the 36-month recovery period. The only arrhythmias noted were premature ventricular contractions and non-sustained ventricular tachycardia in a limited number of patients.

## Discussion

### Main findings

High-intensity, and to a lesser extent low-intensity, chronic VNS is associated with significant, persistent improvements in intrinsic HRR and LVEF, which lasted for the 3-year monitoring period in patients with HFrEF. Because there were parallel and correlated favorable effects on HRT slope, an indicator associated with reduced cardiac mortality, enhanced baroreceptor sensitivity in response to VNS appears to be an underlying physiologic mechanism.

### Prior investigations of cardioprotective actions of VNS

Schwartz, De Ferrari, and others [8, 9, 24] have provided an extensive scientific foundation and encouraging clinical evidence of multiple beneficial effects of VNS in the treatment of heart failure. For example, circulating cytokine levels are increased in patients with HFrEF [25], and VNS has been shown to reduce circulating cytokine levels in an experimental model of impaired LVEF [26]. VNS has also been reported to inhibit neural release of norepinephrine at cardiac effector junctions [27], which in turn normalizes autonomic balance as indicated by improvements in heart rate variability and baroreflex sensitivity [9, 28], attenuates systemic inflammation [29], improves coronary flow [30], and is antiapoptotic [31, 32]. Vagal inhibition of the inflammatory reflex may lead to multifaceted beneficial effects through suppression of macrophage activation, synthesis of tumor necrosis factor, and overall blunting of inflammatory responses [33]. These salutary actions provide scientific underpinnings for clinically documented improvements in cardiac function and reduction in heart failure symptoms [9, 12, 19].

Notwithstanding initial promising results from these preclinical and clinical investigations, the INOVATE-HF trial found that chronic right-sided VNS using the CardioFit™ system failed to decrease mortality or heart failure events [10]. Post hoc analyses suggest that the inability of the INOVATE-HF study to achieve its primary efficacy endpoints may have been due in part to the sizable number of patients with CRT devices [10, 34]. These authors also raised the plausible possibility that the stimulation frequency and intensity were insufficient to achieve autonomic engagement [10, 34]. It is well established that the cardiac response to stimulation is highly dependent on stimulation parameters [35], and the current findings that high- but not low-intensity VNS resulted in beneficial effects on intrinsic HRR, HRT, and LVEF are consistent with this hypothesis.

### Current study

The present investigation constitutes a sub-analysis of the ANTHEM-HF cohort of patients with HFrEF with a mean LVEF of 32.4 ± 7.2%. At variance with the INOVATE-HF study, none of the patients had an ICD or CRT device, and no intracardiac catheter was employed in connection with VNS delivery.

The main finding of this study is that high-intensity chronic VNS was associated with a persistent improvement in intrinsic HRR. A sizable body of evidence indicates that an increase in HRR is associated with favorable cardiovascular outcomes [14, 36–38]. In addition, we found evidence of a significant, enduring increase in HRT slope, a parameter that has been linked to enhanced baroreceptor sensitivity and associated with a decrease in cardiac deaths [18]. The increased baroreceptor sensitivity effect appears to reduce the time constant in intrinsic HRR (Fig. 3, lower panel). The physiologic bases whereby VNS enhances HRT slope are incompletely elucidated. However, a plausible mechanism is that VNS stimulates cervical afferent vagal fibers that innervate the aortic arch baroreceptors. This action in turn, through an influence on the central nervous system cardiovascular regulatory sites, results in a classical reciprocal augmentation of vagal efferent activity and decrease in cardiac-bound sympathetic drive. This sequence is akin to that which occurs when sensory fibers in the aortic arch are stimulated by stretch due to an increase in arterial blood pressure. Interestingly, HRT slope and mean banded relaxation parameter changes appear to be independent of changes in heart rate (*p* = 0.063). Patients enrolled in ANTHEM-HF also experienced improvements in 6-minute walk test and New York Heart Association class at 6, 12, 24, and 36 months [39]. Left ventricular end-systolic diameter and volume did not change across 42 months [40].

VNS was associated with a sustained, robust improvement in LVEF across 3 years. Notably, the increase in LVEF exceeded the ≥ 5% level regarded as a criterion for response status to CRT [41]. The potential mechanisms for improvement in cardiac mechanical function by VNS, based on preclinical studies, include protection of cardiac myocytes through reduction in oxidative stress, decreased apoptosis and inflammatory response, and amelioration of the cardiotoxic consequences of excess levels of catecholamines resulting from muscarinic-receptor-mediated accentuated antagonism.

### Limitations

A limited number of paired AECG recordings from the ANTHEM-HF cohort were available for analysis. However, the present study is strengthened by the inclusion of ~ 2000 heart rate acceleration/deceleration events per 24 h AECG recording for analysis, for a total of ~ 144,000 determinations in the subjects studied across the 3-year period of study. Also, exclusion of patients due to excess ventricular premature beats that precluded analysis of intrinsic HRR or unwillingness to consent to follow-up may have introduced an element of bias in the current results. Thus, there is a need to examine the utility of intrinsic HRR in larger populations of patients in a multinational, randomized, controlled clinical setting to clarify more precisely the role of this parameter in the evaluation and management of HFrEF. In future studies, it may be necessary to modify the intrinsic HRR thresholds somewhat to optimize outcomes. The ANTHEM-HF pilot study was not powered to evaluate the effects of VNS therapy on rehospitalization. The large ANTHEM-HFrEF pivotal study (NCT03425422) will address this question.

### Conclusions

This non-randomized study provides evidence that high-intensity VNS, and to a lesser extent low-intensity VNS,  are associated with a persistent improvement in intrinsic HRR and LVEF that lasts for at least 3 years. Based on the finding that there were corresponding favorable effects on HRT slope, enhanced baroreceptor function in response to VNS may be an underlying physiologic mechanism. These observations carry important scientific and practical clinical implications. Our study emphasizes the need for appropriate VNS parameters and suggests a noninvasive approach for tracking autonomic engagement, which can be evaluated using standard AECG recordings.

## Data Availability

Intrinsic Heart Rate Recovery analytical software is the property of LivaNova USA. The Heart Rate Turbulence software employed for these analyses is available from GE Healthcare (Milwaukee, WI).
